# A Modified Endoscopic Carpal Tunnel Release Surgery

**DOI:** 10.1016/j.jpra.2024.02.015

**Published:** 2024-03-05

**Authors:** Jianyun Yang, Jing Xu, Yinglu Zhao, Xiaotian Jia

**Affiliations:** a Department of Hand Surgery, Huashan Hospital, Fudan University; b Key Laboratory of Hand Reconstruction, Ministry of Health; c Shanghai Key Laboratory of Peripheral Nerve and Microsurgery

**Keywords:** Carpal tunnel syndrome, Median nerve, Endoscopic carpal tunnel release, Surgical technique

## Abstract

In this study, a modified version of the endoscopic carpal tunnel release surgery was introduced, which is safe and easy to handle. Moreover, the requirement for surgical instruments is low. Six patients with carpal tunnel syndrome underwent the modified procedure. No neurovascular injuries occurred in these patients. According to the one-year follow-up data, all the patients were satisfied with the outcomes. The modified endoscopic carpal tunnel release technique has been proven to be safe with satisfactory outcomes in six patients in this study.

## Introduction

Carpal tunnel syndrome (CTS) is a common compression neuropathy of the median nerve arising from increased pressure in the carpal tunnel. Conservative treatments include splinting, neurotrophic drugs, and/or corticosteroid injection.^1^ Surgical treatment is needed if the conservative treatment fails.

Surgical treatments include open carpal tunnel release (OCTR) and endoscopic carpal tunnel release (ECTR). Although there is no consensus on which surgical method is better, ECTR has been widely recognized recently as a safe technique with favorable outcomes when handled by experienced surgeons.^2^^,^^3^

The ECTR technique was first reported by Okutsu in 1987.^4^ Most literature on ECTR deals with Agee single-portal and Chow dual portal techniques.^5^^,^^6^ However, abnormal or aberrant anatomy cannot be identified clearly through endoscopic approaches in certain cases, which can lead to catastrophic complications including injuries to the median nerve and its branches or palmar superficial artery arch.

In this study, a modified version of ECTR was introduced that can be used to avoid the complications due to the abnormal or aberrant anatomy and is easy to handle. According to the one-year follow-up data, all the patients were satisfied with the outcomes.

## Methods

### Indications

The inclusion and exclusion criteria were as follows.

### Inclusion criteria

The patients were diagnosed with CTS according to the symptoms, and results of the physical examinations and nerve conduction studies. The symptoms included numbness or tingling sensation in the median nerve distribution, nocturnal numbness and pain, and weakness of thenar muscle. Physical examinations revealed the presence of thenar muscle atrophy, Tinel's sign, Phalen's test, and reverse Phalen test. Besides, the prolonged latency of the median nerve at the wrist was detected.

### Exclusion criteria

The patients with wrist extension limitation, history of wrist fractures, lesion in the carpal tunnel, and diseases secondary to inflammatory diseases, such as rheumatoid arthritis were excluded.

All patients involved in this study provided their informed consent. Institutional review board approval of our hospital was obtained for this study. All patients in this study were followed up for at least one year.

## Surgical Technique

The surgery was performed under local or regional anesthesia. A tourniquet was applied. The intersection of a longitudinal line along the ulnar border of the middle finger and the Kaplan line could be used to identify an approximate location of the distal end of the transverse carpal ligament (TCL). A transverse incision on the distal wrist crease was made that started from the radial side of the palmaris longus tendon and extended ulnarly for 1-1.5 cm. For the patients without the palmaris longus tendon, a 1-1.5 cm incision centered on the line drawn from the radial border of the ring finger could be made.^7^

**Figure 1:**
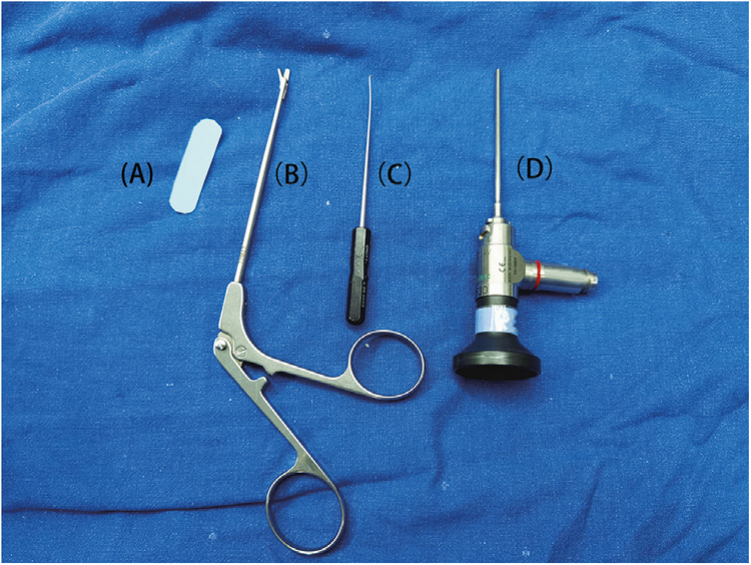
Surgical instruments used in this study: (A) plastic plate; (B) scissors; (C) probe; (D) scope.

The surgical instruments described below are shown in Figure 1.

The median nerve was found at the radial side of the palmaris longus tendon. A mosquito clamp was used to dissect at the superficial layer of the palmar longus tendon from the proximal to distal side. A thin 1.0 cm-wide plastic plate was inserted between the TCL and median nerve from the proximal to distal side. The plastic plate was long enough to reach the Kaplan line. The plate ensured the complete separation of the median nerve from TCL. If the plate could not be inserted easily, abnormal or aberrant anatomy of median nerve might exist. It should be confirmed carefully using an endoscope.

**Figure 2:**
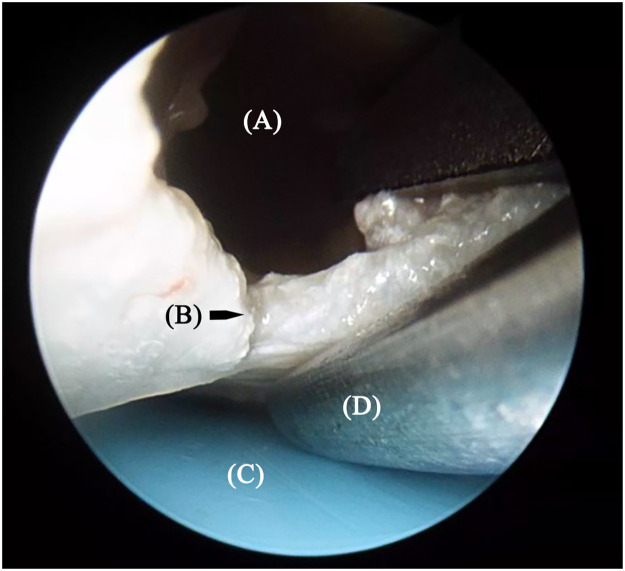
Following the scope, the transverse carpal ligament was cut using the scissors from the proximal to distal end. (A) soft tissue; (B) transverse carpal ligament; (C) plastic plate; (D) scissors.

**Figure 3:**
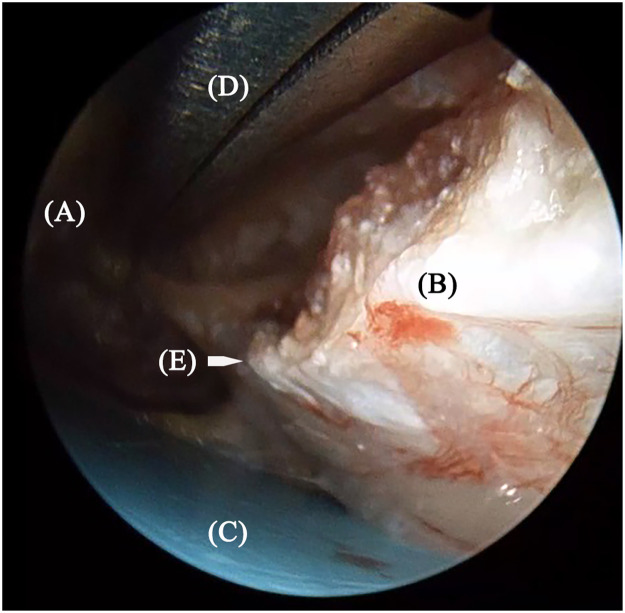
Cutting edge of the transverse carpal ligament, soft tissue over the transverse carpal ligament, and plastic plate could be seen clearly under the scope. (A) soft tissue; (B) transverse carpal ligament; (C) plastic plate; (D) scissors; (E) distal edge of the transverse carpal ligament.

A retractor was used to elevate the skin and fat. The space created by the retractor accommodated the endoscope and wrist arthroscopic scissors. Following the scope, the TCL was cut using the scissors from the proximal to distal side (Figure 2). The cutting edge of the TCL, soft tissue superficial to the TCL, and plastic plate could be seen clearly under the endoscope (Figure 3). The abnormal or aberrant anatomy of the branch of the median nerve could be identified and protected carefully. The distal end of the TCL and fat proximal to the superficial palmar arch could be seen when the release was completed. The proximal antebrachial fascia was then released under direct vision. A probe was introduced into the carpal tunnel from the proximal to distal side. The probe could be felt under the skin if the TCL was completely released. The wound was closed using absorbable sutures. A sterile and soft dressing was applied. Active motion of the digits was encouraged early after surgery.

## Results

The surgical procedure was performed in six patients, five women and one man, who met the inclusion criteria. The age of the patients ranged from 43 to 64 years. The mean age was 52.33 years.

No neurovascular injuries occurred during the procedure in all the six patients. According to one-year follow-up data, all patients completely recovered. However, two of them had pillar pain, which improved with time and completely disappeared in 6 months. All the patients were satisfied with their appearance and short recovery time.

## Discussion

According to the literature,^8^, ^9^, ^10^, ^11^ compared with OCTR, ECTR provided CTS patients with a safer and predictable solution in the hands of well-trained surgeons. The patients could perform their normal activities in a relatively short time with minimal post-operative discomfort.

Agee single-portal technique and Chow dual portal technique were the most used ECTR technique in clinic.^5^^,^^6^ These ECTR surgical techniques were intra-carpal tunnel release. However, abnormal or aberrant anatomy outside the carpal tunnel could not be identified. Our extra-carpal tunnel release technique provided a clear vision inside and outside the carpal tunnel. Neurovascular injuries were avoided. In addition, the endoscope and scissors were not necessarily inserted into the stenotic carpal tunnel before the TCL was cut, which avoided transient neuropraxia. Furthermore, the learning curve of our technique is not steep.

K-Daniel^12^ reported a valuable retractor-endoscopic technique for CTS and cubital tunnel syndrome. Compared with their technique, our technique had several advantages. First, a plate was used to protect the median nerve. Second, abnormal or aberrant anatomy of the median nerve could be identified if the plate could not be inserted easily. Third, the hands of Asian patients are relatively small. A retractor was used to elevate the skin and fat to create a larger space. Fourth, the regular wrist arthroscopic instruments were easily accessible compared with the special instruments used by K-Daniel.

Wing-Yuk^7^ reported another modified surgical technique of ECTR. It was a single-portal technique using instruments originally designed for endoscopic cubital tunnel release. It had the advantage of performing the release with the median nerve protected under direct vision. However, our technique had more advantages. First, the regular wrist arthroscopic instruments used by us are easily accessible. Second, our space was created by blunt dissection at the superficial layer of the palmar longus tendon, which was easy to handle. Third, the space created by the retractor was larger compared with that of Wing-Yuk. The aberrant anatomy of the median nerve branch could be identified easily within the space. Fourth, a plastic plate was used instead of a dissector. The plate is not only thinner but also can spare one hand. However, our study still has some limitations and more clinical cases are needed. Results after long-term follow-up are required.

In conclusion, a modified ECTR technique was performed in 6 patients with satisfactory outcomes.

## Conflicts of interest statement

The authors have no conflicts of interest relevant to this article.
